# Spectrum of Respiratory Viral Illness in the Northwestern Region of Saudi Arabia: A Molecular Multiplex PCR-Based Study in a Tertiary Care Setting

**DOI:** 10.7759/cureus.107372

**Published:** 2026-04-19

**Authors:** Umair Ismail Manghrio, Mesfer Awadh Alyami, Randa Al Shaman, Sultan Al Howeti, Osama Mukhtar

**Affiliations:** 1 Department of Pathology, King Salman Armed Forces Hospital, Tabuk, SAU; 2 Medical Laboratory, King Salman Armed Forces Hospital, Tabuk, SAU; 3 Prince Sultan Oncology Center, King Salman Armed Forces Hospital, Tabuk, SAU

**Keywords:** influenza a virus, multiplex pcr, respiratory syncytial virus, respiratory tract infections, sars-cov-2, seasonal variation, viral coinfection

## Abstract

Background

Respiratory tract infections (RTIs) continue to be a major global health concern, particularly among infants, the elderly, and individuals with chronic illnesses. The circulation patterns of respiratory viruses have shifted considerably after the COVID-19 pandemic, emphasizing the need for updated regional data. This study assessed the distribution, age-related trends, and seasonal behavior of common respiratory viruses in northwestern Saudi Arabia using multiplex PCR diagnostics.

Methods

This retrospective observational study was conducted on 3,809 respiratory samples collected at King Salman Armed Forces Hospital, Tabuk, between November 2022 and April 2025. All specimens were analyzed using the Xpert Xpress CoV-2/Flu/RSV Plus assay (Cepheid, Sunnyvale, CA, USA) to detect SARS-CoV-2, influenza A, influenza B, and respiratory syncytial virus (RSV). Demographic variables, pathogen frequencies, coinfection patterns, and seasonal variations were examined. Associations between age groups and viral detection were evaluated using chi-square and Fisher’s exact tests.

Results

Among 3,809 samples, 1,197 (31%) tested positive for at least one respiratory pathogen. RSV was the most frequently detected virus (39%), followed by influenza A (33%) and SARS-CoV-2 (19%). Single-pathogen infections accounted for 97% of positive cases, while 3% demonstrated coinfections, most commonly RSV plus influenza A and SARS-CoV-2 plus influenza A. Viral detection varied significantly by age (p < 0.001). Children under five years had the highest positivity rate, with RSV dominating this age group. In contrast, influenza A and SARS-CoV-2 were more common among older adults. Seasonal analysis revealed winter peaks for RSV, influenza A, and SARS-CoV-2.

Conclusions

Respiratory viral infections in northwestern Saudi Arabia demonstrate distinct age-related and seasonal patterns, with RSV predominating in young children and influenza A and SARS-CoV-2 more common in older age groups. These findings highlight the importance of continuous molecular surveillance and the role of multiplex PCR diagnostics in guiding clinical management and antimicrobial stewardship. Further prospective studies incorporating clinical severity outcomes are warranted to better assess the public health impact of respiratory viral infections in this region.

## Introduction

Respiratory tract infections (RTIs) are among the most frequent infectious diseases worldwide and contribute substantially to morbidity and mortality. Although all age groups are affected, infants, older adults, and individuals with chronic illnesses are at heightened risk for severe complications such as acute respiratory failure. While several respiratory viruses typically cause mild upper respiratory symptoms, pathogens such as influenza A and B and RSV are responsible for most hospitalizations and deaths associated with viral respiratory disease. The COVID-19 pandemic further altered viral circulation patterns due to widespread public health interventions, including masking, social distancing, and lockdowns [1-5].

International studies have documented varying viral trends during the pandemic. For example, rhinovirus and enterovirus activity remained dominant in Taiwan and Switzerland despite extensive public health measures, whereas influenza activity declined in the same populations [1,3]. Conversely, a study from Turkey documented a marked reduction in influenza cases during the pandemic period, underscoring geographical differences in the patterns of respiratory viral infections [6]. Additionally, findings from a global systematic review indicated that rhinoviruses and enteroviruses continued to circulate persistently and were comparatively less influenced by public health measures implemented during the pandemic than other respiratory viruses [7].

In the Middle East, comprehensive RTI data remain limited. Research from Qatar and Lebanon has indicated that rhinovirus, influenza, and adenovirus are the most frequently detected pathogens [8,9]. In Saudi Arabia, existing studies have largely concentrated on RTIs in children, with RSV and adenovirus being commonly reported as the leading viral agents in pediatric populations [10-14]. However, there is a notable lack of studies examining RTI patterns across all age groups in Saudi Arabia, especially during the COVID-19 pandemic period.

Seasonal fluctuations in respiratory virus activity are well recognized and influenced by environmental conditions, human behavior, and viral characteristics [15]. In temperate climates, influenza and other viruses responsible for the common cold generally show increased activity during the winter months, whereas in tropical regions, their occurrence is more often associated with periods of rainfall [16,17]. Environmental conditions such as lower temperatures and decreased humidity support viral persistence and facilitate transmission, while closer indoor contact among individuals further promotes spread. In addition, influenza viruses and RSV continuously evolve through genetic changes, including antigenic drift and shift, which contribute to recurrent seasonal outbreaks as immunity within the population declines over time [18].

Cultural events such as the annual Hajj pilgrimage in Saudi Arabia may contribute to increased respiratory infection positivity, and the unique climatic conditions of the region may further influence viral transmission. A study conducted among children with RTIs in Riyadh (2013-2014) found viral pathogens in 24% of cases, with RSV being the most common and peaking during the winter season. The findings highlight the substantial contribution of viral etiologies to RTIs and demonstrate a distinct seasonal pattern characterized by higher detection rates in colder months [19]. These observations underscore the importance of ongoing epidemiological monitoring and the implementation of targeted public health strategies, particularly in the context of Saudi Arabia’s hosting of large religious gatherings, which present additional challenges for effective infection control and prevention.

A range of diagnostic approaches is employed to detect pathogens responsible for upper respiratory infections, each characterized by distinct strengths and limitations. Rapid antigen-based assays, for example, provide quick results but often lack sufficient sensitivity [8,9]. In contrast, conventional techniques such as viral culture are generally more sensitive; however, they are time-intensive and may delay clinical decision-making [10]. More recently, multiplex PCR assays have become increasingly utilized in clinical settings due to their ability to deliver rapid and highly accurate identification of multiple respiratory pathogens simultaneously [20].

The GeneXpert platform is an FDA-approved system capable of simultaneously detecting multiple respiratory pathogens with high sensitivity and specificity. The Xpert Xpress CoV-2/Flu/RSV plus assay (Cepheid, Sunnyvale, CA, USA) is a multiplex RT-PCR test designed for the qualitative identification and differentiation of SARS-CoV-2, influenza A, influenza B, and RSV [11-13,18].

This study evaluated the etiological profile and epidemiological patterns of RTIs among patients tested using multiplex PCR at King Salman Armed Forces Hospital (KSAFH) from November 2022 to April 2025. The primary objective was to determine the frequency and distribution of key respiratory viral pathogens, including SARS-CoV-2, influenza, and respiratory syncytial virus (RSV). Secondary objectives included assessing seasonal variation in viral detection (winter: October to March; summer: April to September) and describing pathogen distribution across age groups and gender.

Accurate identification of viral etiologies is essential to guide appropriate clinical management. The use of reliable molecular diagnostic tools can inform treatment decisions, reduce unnecessary antibiotic use, and support antimicrobial stewardship efforts aimed at limiting the development of resistance.

## Materials and methods

Study design and setting

This retrospective observational study was conducted at KSAFH, a 900-bed tertiary care facility under the Ministry of Defense located in Tabuk, Saudi Arabia. Ethical approval for this study was obtained from the KSAFH Research Ethics Team (approval no. KSAFH-RET-2025-643).

Study population and sample collection

A total of 3,809 respiratory specimens were collected from patients presenting with respiratory illness at KSAFH between November 2022 and April 2025. The study cohort was derived from a laboratory-based dataset comprising respiratory samples submitted for diagnostic testing, without stratification by inpatient or outpatient status. Therefore, the analyzed population represents a mixed cohort reflective of routine clinical submissions. Specimens included nasopharyngeal swabs, sputum samples, bronchoalveolar lavage (BAL), and tracheal aspirates. All collected samples were processed using the Xpert Xpress CoV-2/Flu/RSV plus assay on the GeneXpert platform (Cepheid).

Laboratory testing

The Xpert Xpress CoV-2/Flu/RSV plus assay is an automated in vitro diagnostic test designed for the simultaneous qualitative detection and differentiation of RNA from SARS-CoV-2, influenza A, influenza B, and RSV using RT-PCR. The assay is performed on the GeneXpert Instrument Systems (Dx and Infinity platforms; Cepheid). The assay targets specific genomic regions, including the nucleocapsid (N), envelope (E), and RNA-dependent RNA polymerase (RdRP) genes of SARS-CoV-2; influenza A matrix (M), polymerase basic 2 (PB2), and polymerase acidic (PA) genes; influenza B matrix (M) and non-structural (NS) genes; and RSV A and RSV B nucleocapsid genes.

The GeneXpert system integrates automated sample preparation, nucleic acid extraction, amplification, and real-time detection within a single-use, self-contained cartridge, thereby minimizing cross-contamination. Each cartridge includes a sample processing control to ensure adequate sample processing and detect potential RT-PCR inhibitors and a probe check control to verify reagent integrity and proper cartridge function. Specimens (nasopharyngeal swabs, anterior nasal swabs, sputum, BAL, or tracheal aspirates) were collected in viral transport medium, saline, or eNAT™. After gentle mixing, samples were transferred into the cartridge and processed automatically by the GeneXpert system for real-time RT-PCR detection. Published manufacturer data report a sensitivity ranging from 95% to 100% and specificity exceeding 99% for the targeted pathogens. The assay design minimizes cross-reactivity through the use of highly specific primer and probe sets directed at unique genomic regions.

Statistical analysis

All data were compiled and analyzed using IBM SPSS Statistics for Windows, version 26.0 (released 2018; IBM Corp., Armonk, NY, USA). Descriptive analysis was performed to summarize the demographic and clinical variables, which were expressed as counts and percentages.

Associations between categorical variables were evaluated using the chi-square test, particularly for examining relationships such as age group distribution and viral detection, as well as differences in positivity across genders. For seasonal analysis, months were categorized as winter (October to March) and summer (April to September). In instances where the assumptions of the chi-square test were not met due to small expected frequencies, Fisher’s exact test was applied. A p-value < 0.05 was considered statistically significant.

## Results

A total of 3,809 respiratory samples were included in the study. The proportion of male patients (55.3%, n = 2,105) was slightly higher than that of female patients (44.7%, n = 1,704). The majority of samples were obtained from children aged 1-5 years (25.6%), and cumulatively, more than 41% of samples were from patients aged ≤5 years. Approximately 19% of samples were from patients aged ≥65 years. The detailed demographic characteristics of the study population are presented in Table 1.

**Table 1:** Demographic characteristics of the study population This table presents the demographic distribution of the study population (n = 3,809), including gender and age group categories. Data are reported as frequencies and percentages. Age was stratified into five groups (≤1 year, 1-5 years, 6-17 years, 18-64 years, and ≥65 years) to facilitate subgroup analysis. These baseline characteristics provide context for interpreting the distribution of respiratory viral infections across the population.

Demographic characteristics		Frequency (n)	Percentage (%)
Gender	Male	2105	55.3%
	Female	1704	44.7%
Age group	≤1 year	602	15.8%
	1-5 years	974	25.6%
	6-17 years	699	18.4%
	18-64 years	815	21.4%
	≥65 years	719	18.9%

Multiplex PCR findings

Out of all the specimens analyzed, 1,197 were positive for at least one respiratory viral pathogen, corresponding to an overall positivity rate of 31%. This finding suggests that approximately one in three tested samples showed evidence of a detectable respiratory infection, highlighting a considerable circulation of viral pathogens within the study cohort.

**Figure 1:**
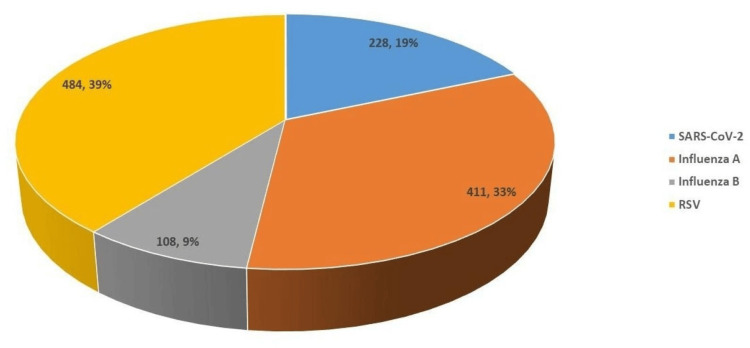
Distribution of respiratory viral pathogens detected by multiplex PCR This figure illustrates the number and proportion of respiratory viral pathogens identified among positive samples (n = 1,197) using multiplex PCR testing. The pathogens displayed include RSV, influenza A, SARS-CoV-2, and influenza B. The graphical representation allows comparison of pathogen prevalence, with each bar (or segment) corresponding to the frequency of detection. RSV, respiratory syncytial virus

With respect to pathogen distribution, RSV was identified as the leading cause, with 484 cases comprising 39% of all positive results. Influenza A ranked next in frequency, being detected in 411 cases (33%), while SARS-CoV-2 accounted for 228 cases (19%). In contrast, influenza B was observed less commonly, with 108 documented cases. A detailed representation of the relative frequencies and distribution of these pathogens is provided in Figure 1.

Number of viruses per sample

Among the 1,197 samples that yielded positive results, the overwhelming majority were characterized by the detection of a single respiratory virus. Specifically, 1,163 cases (97%) involved mono-viral infections, underscoring that respiratory illnesses within this cohort were predominantly attributable to a single etiologic agent.

**Figure 2:**
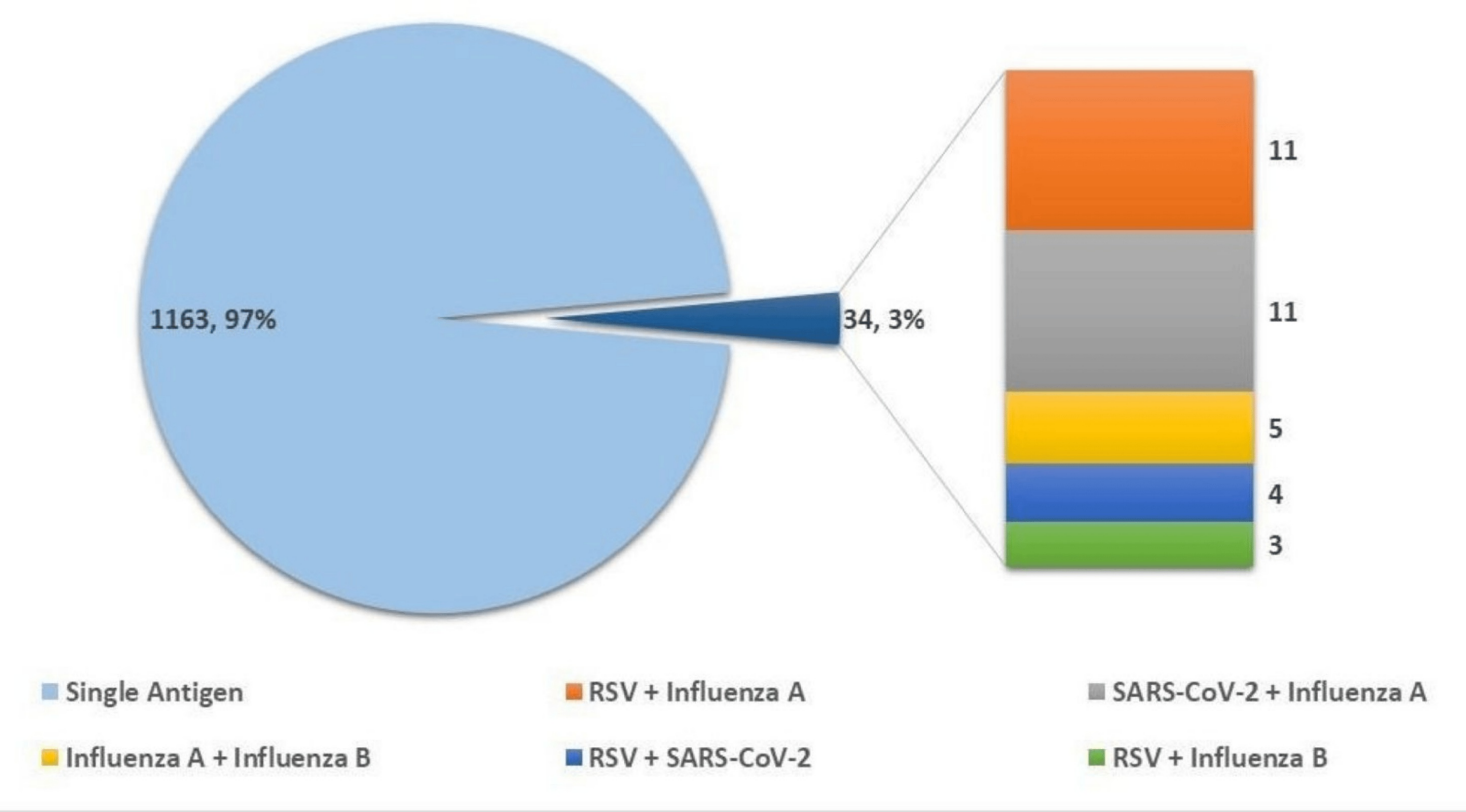
Distribution of the number of viruses detected per sample This figure illustrates the distribution of mono-viral infections and coinfections among positive samples based on multiplex PCR results. Mono-infections (n = 1163; 97%) represent samples with a single detected pathogen, whereas coinfections (n = 34; 3%) indicate the presence of two viral pathogens within the same sample. The figure provides a comparative visualization of the frequency of single versus multiple viral detections. RSV, respiratory syncytial virus

A comparatively small subset of cases, comprising 34 samples (3%), demonstrated the presence of dual viral pathogens, consistent with coinfection. Analysis of these co-infected cases revealed that the most frequently observed viral combinations were RSV with influenza A and SARS-CoV-2 with influenza A, each identified in 11 instances. This pattern suggests a notable tendency for influenza A to co-occur with other respiratory viruses in a minority of patients. The overall distribution of monoviral and coinfection cases is illustrated in Figure 2.

Seasonal variation in viral detection

A statistically significant seasonal variation was observed in the distribution of respiratory viruses across the study period. The majority of detected viruses demonstrated a marked predominance during the winter months (October to March), with significantly higher detection rates compared to the summer months (April to September). This trend was consistent across all major viral pathogens identified in the RTI cohort. Statistical analysis confirmed a strong association between viral detection and seasonality, with all viruses showing highly significant differences (p < 0.001). In contrast, comparatively lower frequencies were observed during the summer season, indicating a clear seasonal pattern of viral circulation favoring colder months.

Association between PCR findings and gender

When comparing multiplex PCR positivity between genders, positive cases were slightly higher among males (n = 642, 54%) than females; however, this difference was not statistically significant (p > 0.05). Similarly, no statistically significant differences were observed between males and females when individual pathogens were analyzed separately (p > 0.05).

Association between PCR findings and age groups

A statistically significant relationship was identified between age group and the overall detection of respiratory pathogens, as demonstrated by the chi-square analysis (χ² = 44.85, p < 0.001). This finding indicates that the likelihood of testing positive for a respiratory virus varied meaningfully across different age categories. Additional analyses further confirmed that this variation was not limited to overall positivity but extended to each individual pathogen, with all comparisons reaching statistical significance.

Specifically, significant differences across age groups were observed for SARS-CoV-2 (χ² = 61.21, p < 0.001), influenza A (χ² = 39.31, p < 0.001), influenza B (χ² = 21.69, p < 0.001), and RSV (χ² = 364.69, p < 0.001). These results highlight distinct age-related patterns in the distribution of respiratory viral infections. The highest overall positivity rate was recorded among children aged five years or younger, suggesting an increased susceptibility or exposure within this age group.

Further examination of pathogen-specific trends revealed that RSV was predominantly detected in children aged ≤5 years, reinforcing its well-established association with younger pediatric populations. In contrast, SARS-CoV-2 and Influenza A were more frequently identified among older individuals, indicating differing epidemiological profiles across age strata. A comprehensive summary of the chi-square analyses and corresponding statistical findings is provided in Table 2.

**Table 2:** Association between age groups and respiratory viral detection using chi-square analysis This table summarizes the distribution of overall and pathogen-specific PCR positivity across predefined age groups. The number of positive cases for SARS-CoV-2, influenza A, influenza B, and RSV is presented for each category. The chi-square test was used to evaluate the association between age groups and viral detection. Chi-square values and corresponding p-values are reported, with statistical significance defined as p < 0.05. This table highlights age-related differences in the prevalence of respiratory viral infections. RSV, respiratory syncytial virus

Age group	Overall positive pathogen	SARS-CoV-2	Influenza A	Influenza B	RSV
≤1 year	242	32	30	7	179
1-5 years	342	29	90	31	205
6-17 years	185	22	81	31	54
18-64 years	233	70	120	30	21
≥65 years	195	75	90	9	25
Chi-square value	44.85	61.21	39.31	21.69	364.69
p-value	0.00	0.00	0.00	0.00	0.00

Association between viral coinfections and age groups

Fisher’s exact test was applied to evaluate the relationship between specific viral coinfection patterns and age group distribution. The analysis demonstrated that the occurrence of combined viral infections was not uniform across age categories, indicating a meaningful variation in coinfection trends among different age groups.

More specifically, coinfection involving RSV and SARS-CoV-2 was observed more frequently in older individuals, whereas the combination of RSV and influenza A was predominantly identified in children aged five years or younger. These contrasting patterns suggest that the interaction between circulating respiratory viruses may differ according to age-related susceptibility and exposure dynamics.

**Figure 3:**
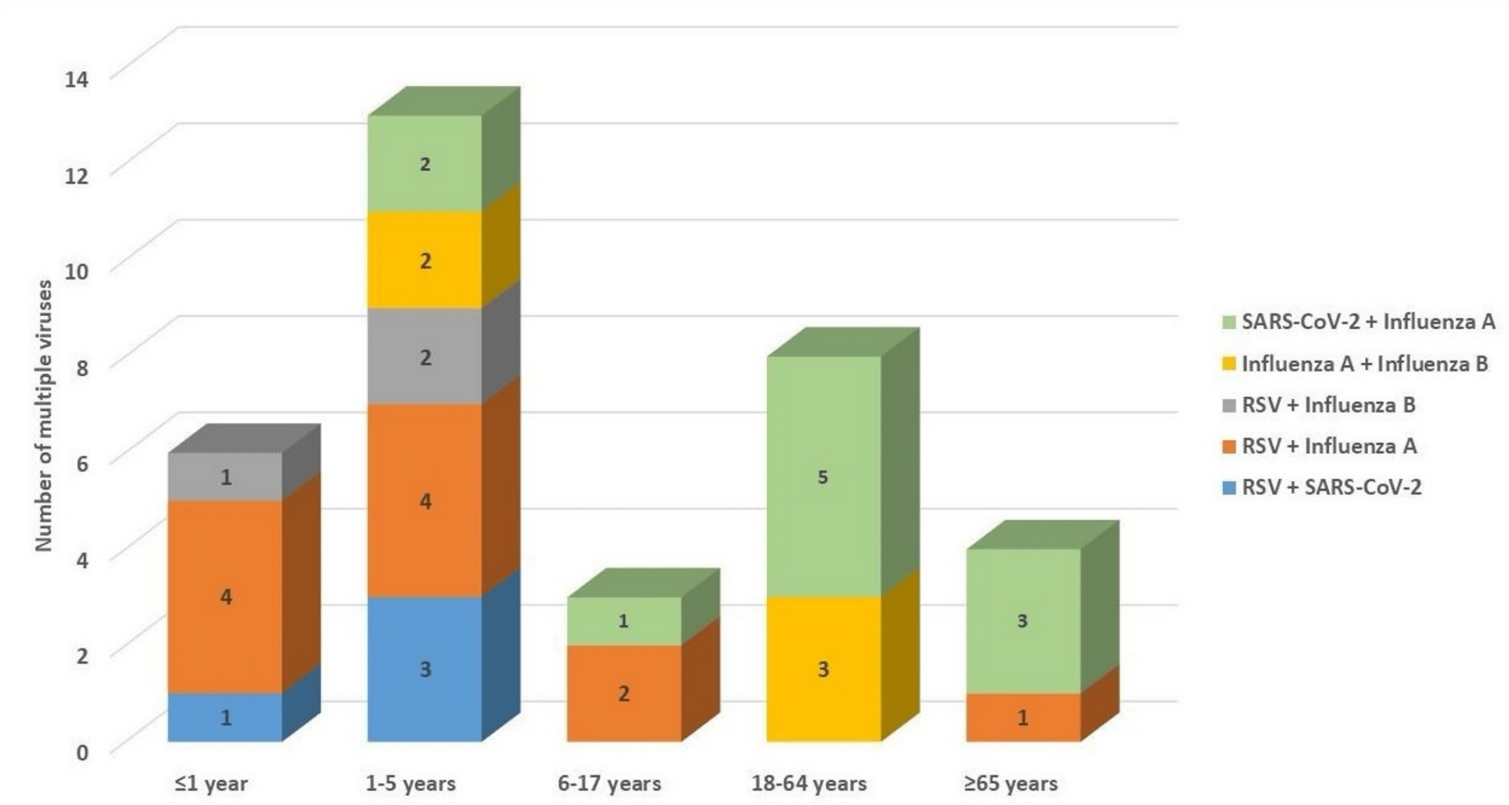
Distribution of viral coinfection patterns across age groups This figure depicts the distribution of viral coinfection combinations across different age groups, as identified by multiplex PCR testing. The primary coinfection patterns include RSV with SARS-CoV-2 and RSV with influenza A. Fisher’s exact test was applied to assess the association between coinfection type and age group (Fisher’s exact = 20.79, p = 0.039). The figure illustrates variations in coinfection patterns between pediatric and older populations. RSV, respiratory syncytial virus

The overall association between coinfection type and age group reached statistical significance, as indicated by Fisher’s exact test (value = 20.79, p = 0.039). A visual summary illustrating the distribution of these coinfection patterns across age groups is provided in Figure 3.

## Discussion

RTIs continue to represent a major cause of morbidity and mortality globally, affecting individuals across all age groups. Nonetheless, certain populations, including young children under five years, older adults, and individuals with preexisting comorbidities, are particularly susceptible to severe disease outcomes. In the present study, respiratory viral infections were detected in 1,197 (31%) of 3,809 tested patients, with 97% of positive specimens harboring only a single viral pathogen. These findings align with previously reported global prevalence rates, which range from 43% to 95%, and are consistent with prior studies in Saudi Arabia, which reported rates between 48% and 92% [10-14]. The variation in reported prevalence across studies may reflect differences in the populations studied, clinical characteristics, presence of coinfections, genetic predispositions, the number of viruses included, and the diagnostic methods employed [21-23].

Age-specific analysis revealed that the highest viral positivity was observed in children aged 1-5 years (25.6%), followed by adults aged 18-64 years (21.4%), the elderly population aged ≥65 years (18.9%), and school-aged children and adolescents aged 6-17 years (18.4%). Infants under one year showed lower positivity. Increased susceptibility among younger age groups may be due to immature immune responses, declining maternal antibodies after six months of age, suboptimal hygiene, and higher exposure to pathogens [22-24]. Additionally, higher infection rates in infants may reflect parents’ prompt healthcare-seeking behavior for symptomatic children. Consistent with earlier reports, a male predominance was observed in acute RTIs, with boys accounting for 59.5% of cases [22,24]. This sex-based difference may reflect immunological, hormonal, or behavioral factors that predispose males to respiratory viral infections during childhood. Among single-pathogen infections, RSV was the most frequently detected virus (39%), followed by influenza A virus (33%) and SARS-CoV-2 (19%). These findings align with prior studies from Saudi Arabia and other regions that consistently identify RSV and influenza A as leading causes of viral respiratory infections, particularly in pediatric and elderly populations [10,12,19].

Multiple viral positivity was identified in 3% of patients with confirmed respiratory viral infections, indicating coinfection with more than one respiratory pathogen. Although the overall proportion of coinfections was relatively low, the observed viral combinations are epidemiologically and clinically significant. In this study, RSV-SARS-CoV-2 coinfection was predominantly observed among elderly patients, whereas RSV-influenza A coinfection was more common among younger individuals.

Coinfections involving RSV and SARS-CoV-2 have been increasingly reported since the COVID-19 pandemic, particularly in older adults and individuals with comorbidities. Advanced age is associated with immunosenescence, impaired mucociliary clearance, and chronic underlying conditions, all of which increase susceptibility to simultaneous viral infections and may exacerbate disease severity [4,5].

In contrast, RSV-influenza A coinfection was more frequently detected among children and adolescents in our cohort. This observation aligns with previous pediatric studies demonstrating that young children are particularly vulnerable to viral coinfections due to immature immune responses, high exposure in school or daycare settings, and frequent circulation of RSV and influenza during the same seasonal periods [14,24].

The relatively low prevalence of viral coinfections observed in this study may reflect viral interference phenomena, whereby infection with one respiratory virus transiently reduces susceptibility to secondary viral infections through innate immune mechanisms, particularly interferon-mediated responses [25]. Globally, RSV remains a major cause of acute lower respiratory infections and hospitalization among young children, contributing substantially to disease burden and mortality worldwide [26].

Seasonal patterns were evident, with respiratory infections peaking during the colder months. RSV, influenza A, and SARS-CoV-2 showed marked winter seasonality. These findings are consistent with international reports, such as those from Greece, which documented winter peaks for influenza A and adenovirus [27]. The US CDC similarly notes that RSV activity traditionally peaks in winter, a pattern temporarily disrupted by the COVID-19 pandemic but later returning to typical seasonal cycles [28]. Surveillance in the United Kingdom has also documented increased RSV circulation during winter [29].

A review by Suryadevara et al. further highlights that influenza, human coronaviruses, and RSV generally peak in winter, whereas viruses such as rhinovirus, adenovirus, and human metapneumovirus circulate throughout the year [24]. Our findings correspond with these observations, showing winter peaks for influenza and RSV. Notably, RSV activity in our cohort began earlier (October) and declined by February, differing from a 2013-2014 Saudi study, which reported peaks from December to March [19]. This discrepancy may be attributed to differences in diagnostic methods (multiplex PCR vs. DFA), improved surveillance, increased public awareness, and better healthcare access, all of which can influence the timing and intensity of viral epidemics. The observed winter peaks are likely influenced by Saudi Arabia’s arid climate, characterized by extreme temperatures and low humidity. Reduced humidity during colder months may enhance viral stability and facilitate airborne transmission, while increased indoor gatherings during winter likely contribute to the spread of respiratory viruses. Future research incorporating meteorological data alongside infection rates could provide a more comprehensive understanding of factors influencing viral transmission.

The importance of this study lies in its detailed analysis of respiratory pathogen distribution and seasonality during a period when global health systems were primarily focused on managing COVID-19. To our knowledge, this represents one of the largest investigations in the Middle East reporting respiratory pathogen prevalence using FilmArray multiplex PCR technology, offering insights that differ from pre-pandemic patterns [1,7,30].

This study is subject to certain limitations. Being a retrospective, single-center, laboratory-based analysis using a multiplex PCR platform, it did not capture clinical information such as symptom severity, length of hospitalization, ICU admission, or mortality outcomes. Furthermore, differentiation between inpatient and outpatient status was not available in the laboratory database. The diagnostic panel was limited to pathogens included in the GeneXpert respiratory assay, which did not allow detection of bacterial or fungal coinfections beyond the tested viral targets. Despite these limitations, the large sample size of over 3,809 specimens enhances the robustness of the findings. Future prospective multicenter studies integrating detailed clinical data are warranted to provide a more comprehensive assessment of the burden of respiratory infections in this population.

## Conclusions

This study offers an in-depth analysis of the epidemiology and seasonal patterns of respiratory viral infections within a tertiary care hospital in northwestern Saudi Arabia. RSV was the most frequently detected pathogen, followed by influenza A and SARS-CoV-2, with significant age-related differences observed in pathogen distribution. Viral infections were more common among children aged ≤5 years, while SARS-CoV-2 and influenza A were more prevalent in older age groups. Clear seasonal trends were identified, with peaks occurring during the winter months.

These findings underscore the importance of continuous molecular surveillance, age-targeted prevention strategies, and the use of rapid multiplex PCR diagnostics to support appropriate clinical management and antimicrobial stewardship. Further prospective research that incorporates clinical severity indicators is necessary to more accurately assess the public health burden of these infections in this population.
